# Non-negative connectivity causes bow-tie architecture in neural circuits

**DOI:** 10.3389/fncir.2025.1574877

**Published:** 2025-08-18

**Authors:** Zhaofan Liu, CongCong Du, KongFatt Wong-Lin, Da-Hui Wang

**Affiliations:** ^1^Peking University HuiLongGuan Clinical Medical School, Beijing Huilongguan Hospital, Beijing, China; ^2^School of Systems Science, Beijing Normal University, Beijing, China; ^3^Intelligent Systems Research Centre, School of Computing, Engineering and Intelligent Systems, Ulster University, Londonderry, United Kingdom; ^4^National Key Laboratory of Cognitive Neuroscience and Learning, Beijing Normal University, Beijing, China

**Keywords:** bow-tie architecture, neural circuits, non-negative connectivity, computational neuroscience, robustness, efficiency, discrimination tasks, backpropagation algorithm

## Abstract

Bow-tie architecture (BTA) is widely observed in biological neural systems, yet the underlying mechanism driving its spontaneous emergence remains unclear. In this study, we identify a novel formation mechanism by training multi-layer neural networks under biologically inspired non-negative connectivity constraints across diverse classification tasks. We show that non-negative weights reshape network dynamics by amplifying back-propagated error signals and suppressing hidden-layer activity, leading to the self-organization of BTA without pre-defined architecture. To our knowledge, this is the first demonstration that non-negativity alone can induce BTA formation. The resulting architecture confers distinct functional advantages, including lower wiring cost, robustness to scaling, and task generalizability, highlighting both its computational efficiency and biological relevance. Our findings offer a mechanistic account of BTA emergence and bridge biological structure with artificial learning principles.

## 1 Introduction

Bow-tie architecture (BTA) is ubiquitous in biological systems (Csete and Doyle, 2004; Tieri et al., 2010; Hilliard et al., 2023). BTA features a markedly smaller or simpler intermediate system that links much larger and more complex upstream and downstream components. This structural arrangement enables BTA to streamline complex interactions and allow modular control in biological processes. Consequently, it has inspired research into its potential applications in synthetic gene circuits (Prochazka et al., 2014) and immune regulation (Carrión et al., 2023). In the context of information processing, the intermediate “waist” of the architecture integrates diverse upstream inputs into a compact representation, which is then reused to generate a wide range of downstream outputs. But the the underlying mechanisms of its emergence are still unclear.

**Figure 1:**
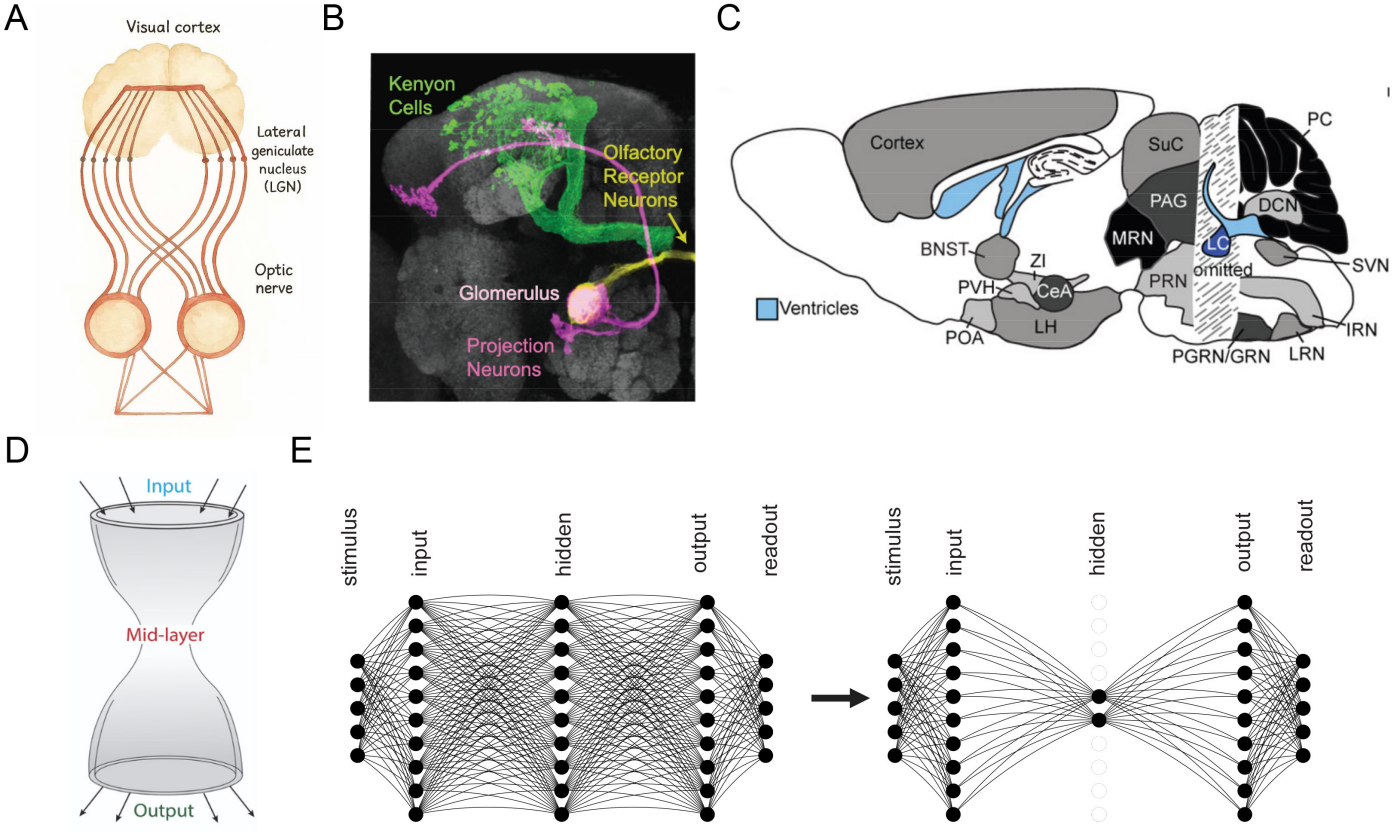
Observed and emergent bow-tie architecture (BTA) in neural networks. **(A–C)** BTA observed in visual **(A)**, olfactory **(B)**, and neuromodulatory **(C)** systems. **(A)** redrawn from Nicholls et al. (2001). [**(B,C)** reproduced with permission from Caron et al. (2013); Schwarz et al. (2015)]. **(D)** Schematic of BTA or “hourglass” structure (Friedlander et al., 2015). **(E)** Transformation of a network from without BTA (**left**) to BTA (right) through training.

BTA is evident across various neural circuits, typically characterized by a smaller ensemble of neurons within a specific brain region that receives converging input from, and projects diverging output to, larger populations of neurons distributed across broader brain systems (Figures 1A–C). This forms an hourglass-like structure (Figure 1D). A well-known example of BTA is the mammalian visual pathway. Retinal ganglion cells project to the lateral geniculate nucleus (LGN) in the thalamus, which acts as a functional bottleneck by filtering and transforming visual signals before relaying them to the primary visual cortex (V1). In V1, the information is then distributed widely across a large population of cortical neurons (Zhaoping, 2006; Usrey and Alitto, 2015; Hammer et al., 2015; Morgan et al., 2016; Rompani et al., 2017; Rosón et al., 2019; Jang et al., 2020) (Figure 1A). Similarly, the Drosophila olfactory system exhibits a BTA with a convergence-divergence organization. Approximately 40 olfactory receptor neurons (ORNs) per receptor type project to a single glomerulus in the antennal lobe (Laurent, 2002), which connects to a small number of projection neurons (PNs), typically 3–5 per glomerulus (Turner et al., 2008; Dhawale et al., 2010; Singh et al., 2019). These PNs then diverge to innervate thousands of Kenyon cells (KCs) in the mushroom body (Caron et al., 2013), with each KC sampling input from 7 randomly selected PNs, resulting in a substantial expansion phase (Figure 1B). Beyond sensory systems, the thalamus itself is known to act as a middleman-like processor in large-scale cortico-thalamic-cortical loops, mediating information flow between widespread cortical areas (Sherman and Usrey, 2021; Worden et al., 2021; Shepherd and Yamawaki, 2021). Another prominent example involves chemical neuromodulatory systems, including the relatively small midbrain dopaminergic nuclei, raphe serotonergic nuclei, and the noradrenergic locus coeruleus, which receive diverse afferent inputs and project widely across the brain (Beier et al., 2015; Kohl et al., 2018; Luo, 2021; Sabrin et al., 2020) (Figure 1C).

It has been shown that BTA, or its partial implementation, contributes to efficient information processing by improving information retention (Gutierrez et al., 2021), enhancing computational speed and accuracy (Jeanne and Wilson, 2015), and amplifying stimulus variability while enhancing representational capacity (Babadi and Sompolinsky, 2014). BTA has been applied in engineering domains such as face recognition (Hammouche et al., 2022) and emotion recognition (Cheng et al., 2024). In machine learning, BTA is widely adopted in autoencoders, where a low-dimensional latent representation at the bottleneck layer enables feature compression and dimensionality reduction (Bourlard and Kamp, 1988). Despite the increasing awareness of BTA's functional roles in biological and artificial systems, the underlying mechanisms of its spontaneous emergence in neural system remain poorly understood.

Numerous theoretical studies have explored the emergence of bow-tie architectures (BTA) in the context of biological networks such as metabolic, gene regulatory, and signaling systems, rather than in neural systems (Friedlander et al., 2015; Itoh et al., 2024) (Figure 1D, right). For instance, (Friedlander et al. 2015) employed linear transformation within network models and demonstrated that BTA structures can naturally evolve when information compression is beneficial for achieving low-dimensional output goals. Similarly, Tishby et al. (2000) applied principles from information theory to explain how BTAs can emerge as efficient solutions to input-output mapping problems under certain constraints. These studies provided foundational insights into how selective constraints and informational objectives may drive the formation of modular, compressive network motifs.

Neural networks in both neuroscience and machine learning are inherently nonlinear, and weights evolve through learning algorithms aimed at task performance rather than matrix matching (Rumelhart et al., 1986; Yamins and DiCarlo, 2016; Yang et al., 2019). Moreover, previous neural models often relied on manually specified architectures, lacking the generality required to explore spontaneous BTA formation. From a neurobiological perspective, long-range corticocortical projections are predominantly excitatory, mediated by glutamatergic synapses (Wang, 2025). For example, pyramidal neurons in cortical layers 3 and 5 project to other cortical and subcortical areas via excitatory pathways (Douglas and Martin, 2004). In sensory hierarchies, feedforward connections are largely excitatory, facilitating the progressive integration of sensory information (Wang et al., 2021; Li, 2014). In contrast, inhibitory interneurons are typically local and are less involved in long-range inter-areal communication (Markram et al., 2004).

Despite the widespread presence of bow-tie architectures (BTAs) in biological systems, their spontaneous emergence in neural circuits remains poorly understood due to several key research gaps. First, prior computational studies have largely relied on linear models or manually specified architectures (Friedlander et al., 2015; Tishby et al., 2000), limiting their ability to capture the self-organizing dynamics of real neural systems. Second, these models often ignore the inherent nonlinearity of neural computation, where weights evolve through learning algorithms aimed at task performance rather than analytical matrix construction (Rumelhart et al., 1986; Yamins and DiCarlo, 2016; Yang et al., 2019). Third, most existing frameworks lack biological plausibility, overlooking fundamental anatomical constraints, such as the dominance of excitatory glutamatergic long-range projections in cortical hierarchies (Wang, 2025; Douglas and Martin, 2004). These omissions hinder our ability to explain how BTAs might naturally arise in learning-driven, biologically constrained systems.

To address these limitations, we investigate whether biologically inspired constraints, specifically excitatory (which is non-negative) feedforward connectivity, can drive the spontaneous emergence of bow-tie architecture (BTA) in artificial neural networks. In particular, we examine how non-negativity shapes architectural compression during learning and assess the potential computational advantages conferred by such emergent structures, including wiring efficiency, enhanced robustness, and structural stability. Additionally, we explore the generality of BTA emergence across diverse classification tasks and varying output dimensionalities.

Thus, our study aims to uncover the mechanistic role of non-negative connectivity in the spontaneous emergence of bow-tie architecture (BTA) in neural circuits. Specifically, we investigate whether biologically inspired constraints, particularly the prevalence of non-negative feedforward connections–can drive the self-organization of compressive structures in neural networks. We train feedforward architectures with non-negative weights on a range of classification tasks using publicly available datasets to test this hypothesis (Figure 1E). Our goal is to evaluate whether non-negativity facilitates spontaneous architectural compression, and to assess the computational advantages of the resulting BTA, including stability with increasing output dimensionality, reduced wiring cost and robustness across datasets .

## 2 Research methodology

### 2.1 Data description

Three datasets were used for the classification tasks. For the first dataset, we synthetically generated a total of 2 million samples. Each sample *s* = (*s*_1_, *s*_2_, ⋯ , *s*_50_) is composed of signal *x* and noise ϵ, i.e. *s*_*i*_ = *x*_*i*_+ϵ_*i*_, where *x*_*i*_~*U*(0, 1) and ϵ_*i*_~*N*(0, 0.05). For each stimulus *s*, we generated 20, 000 samples, and we divided them into 100 categories or classes based on the mean value of the samples.

To verify the robustness of the BTA's performance, we used two other open datasets. One of them is the well-established MNIST (LeCun et al., 2002) with 60, 000 samples of handwritten digits. The other is the odor detection data (Wang et al., 2021), where each odor is denoted as *s* = (*s*_1_, *s*_2_, ⋯ , *s*_50_) and *s*_*i*_~*U*(0, 1) with a total of 1, 008, 192 samples, and the samples were categorized into 100 classes according to their nearest neighbors (Cover and Hart, 1967).

### 2.2 Neural network model and architecture

Feedforward neural network model architecture was used in this study. The neurons were distributed in 5 layers, namely, they are the stimulus, input, hidden, output, and readout layers (Figure 1E, left). The input layer of neurons represents sensory-based neurons, such as the retina in the visual system or neurons with olfactory receptors in the olfactory system. The hidden layer of neurons can be considered as an intermediate processing stage, similar to the ganglion cells in the visual system or projection neurons in the olfactory system. The output layer of neurons represents the primary visual cortical neurons or Kenyon cells in the visual or odor systems, respectively. The readout layer of neurons encodes the classes, with the number of readout neurons equaling the number of classes to be discriminated or identified by the model.

The activity of each layer was represented by a vector as *h*_0_, *h*_1_, *h*_2_, *h*_3_ and *h*_4_ for the respective 5 layers in the network. Each activity vector had a dimension 1 × *n*_*i*_ where *n*_*i*_ indicated the number of neurons in the *i*^*th*^ layer. We let matrix *W*_1_ describe the connections from the stimulus layer to the input layer, and *W*_2_, *W*_3_ and *W*_4_ for connections from the input to the hidden, from the hidden to the output, and the output to the readout layer, respectively. For simplification, we set *h*_0_ = *s* where *s* is the stimulus. We used the nonlinear rectified linear unit (ReLU) function to describe the activity of the input, the hidden, the output, and the readout neurons, mathematically described by $h_{i}=ReLU\left(W_{i}^{T}h_{i-1}+b_{i}\right)$, where *i* = 1, 2, 3, 4, and *b*_*i*_ is a bias vector.

### 2.3 Model training and testing

The network was initialized as a fully connected network and the initial connection weights followed a uniform distribution between 0.01 and 0.2. We used a standard backpropagation (BP) algorithm (Rumelhart et al., 1986) to train the network to classify the stimuli into different classes, with support regarding its approximation in neurobiological systems (Whittington and Bogacz, 2019). We chose cross entropy as the loss function (de Boer et al., 2005): $Loss=-\underset{j}{\sum }y_{j}log\left(p_{j}\right)$, with $p_{j}=exp\left(h_{4}\left(j\right)\right)/\left(\overset{N}{\underset{k}{\sum }}exp\left(h_{4}\left(k\right)\right)\right)$, where *y* is the predefined label of stimulus in vector form, *p*_*j*_ the soft-max function of the *j*^*th*^ readout neuron. The connection weights for consecutive iterations were updated as follows:


(1) $$\begin{array}{l} W_{4}\leftarrow W_{4}-\eta h_{3}^{T}\left(p-y\right) \end{array}$$

(2) $$\begin{array}{l} W_{3}\leftarrow W_{3}-\eta h_{2}^{T}\left(p-y\right)W_{4}^{T} \end{array}$$

(3) $$\begin{array}{l} W_{2}\leftarrow W_{2}-\eta h_{1}^{T}\left(p-y\right)W_{4}^{T}W_{3}^{T} \end{array}$$

(4) $$\begin{array}{l} W_{1}\leftarrow W_{1}-\eta s^{T}\left(p-y\right)W_{4}^{T}W_{3}^{T}W_{2}^{T} \end{array}$$

where η = 0.1 is the learning rate. We followed the stochastic gradient descent method (Bottou et al., 2016) and used the Keras Adam optimizer to train the network (Kingma and Ba, 2014). In the presence of non-negative constraint, *W*_1_, *W*_2_ and *W*_3_ were set to be non-negative constrain,namely, with *W* = 0 if *W* < 0.

### 2.4 Model parameters and performance on three tasks

To assess the specific effect of the non-negative constraint, we compared models trained with and without this constraint across three datasets. As shown in Tables 1, 2, while unconstrained models achieved marginally higher accuracy, they exhibited significantly denser connectivity, higher wiring cost, and lacked the bow-tie architecture. In contrast, non-negative models naturally formed a bottleneck in the hidden layer, with sparse activation and reduced wiring cost, supporting our hypothesis that non-negativity drives structural emergence of bow-tie organization.

**Table 1:** Training details for three tasks with non-negative constraint.

	**Synthetic dataset**	**MNIST dataset**	**Odor detecting dataset**
No. classes	100	10	100
Input dimension	50	784	50
Batch size	256	512	256
No. epochs	20	200	20
Training set size	600,000	36,000	1,000,000
Testing set size	400,000	24,000	8,192
Initial neurons	500, 500, 500	1,000, 1,000, 1,000	500, 500, 2,500
Convergence rate	0.850	0.307	-0.318
Accuracy of training set	99.57%	96.13%	82.48%
Accuracy of testing set	99.55%	92.60%	78.65%
No. active neurons	225.69, 153.31, 498.85	380.14, 87.92, 997.76	62.08, 48.97, 2,308.74
F-1 score	0.9699	0.9707	0.7436
Sparsity (%)	75.4	81.7	89.6
Bottleneck ratio	0.31	0.11	0.10
Wiring cost	16,372.4	20,639.2	13,492.7
Bow-tie formed?	Yes	Yes	Yes

The data for the training results is based on the average of 50 training sessions.

**Table 2:** Training details for three tasks without non-negative constraint.

	**Synthetic dataset**	**MNIST dataset**	**Odor detecting dataset**
Convergence rate	0.825	0.402	-0.382
Accuracy of training set	99.84%	99.72%	77.85%
Accuracy of testing set	99.62%	99.53%	76.56%
No. active neurons	432.13, 426.87, 428.92	805.24, 884.14, 853.76	470.83, 464.74, 2,292.46
F-1 score	0.9642	0.9785	0.7601
Sparsity (%)	22.7	15.3	12.6
Bottleneck ratio	0.97	0.98	0.95
Wiring cost	42385.1	50614.7	73482.5
Bow-tie formed?	No	No	No

The data for the training results is based on the average of 50 training sessions. Number of classes, input dimension, batch size, number of epochs and set size are the same as those in Table 1.

To further evaluate the robustness of this effect, we conducted a comprehensive sensitivity analysis across multiple random seeds, network sizes, and pruning conditions. As detailed in the Supplementary Section S1, the emergence of bow-tie architecture and associated performance advantages remained consistent across all tested configurations. In particular, we observed stable accuracy, reproducible sparsity patterns–especially in the second hidden layer–and strong resilience of bow-tie structure even under architectural perturbations.

In addition, to assess the generalization capacity of the model beyond the training distribution, we performed out-of-distribution (OOD) tests using perturbed input data (see Supplementary Section S2). The model maintained high accuracy under additive Gaussian noise ( 86% ), but showed performance degradation when 30% of the input features were occluded (accuracy dropped to 50%). This asymmetry highlights the model's robustness to unstructured noise, while also suggesting reliance on a sparse set of informative features–consistent with the compressive nature of the bow-tie structure.

These results collectively underscore the statistical rigor, generalizability, and biological plausibility of our findings.

### 2.5 Data analysis

We recorded the activity of each neuron exposed to different samples and calculated the mean and variance of the activity over the samples. The active neuron are defined as its standard deviation below 0.01. To investigate the effects of non-negative connectivity constraints, we trained 10 networks (training more networks yielded similar results) with non-negative connectivity constraint given different initialized connection weights to perform the same classification task. We then trained another 10 network without a non-negative connectivity constraint to perform the same classification task. After training, we compared the connectivity of these networks and the activity of neurons.

The source codes, generated data, and analyses that support the findings of this study will be made available upon publication.

## 3 Results

### 3.1 Emergence of bow-tie architecture in neural network

To demonstrate the emergence of BTA, we first trained a five-layer neural network, with 500 neurons in each layer on a generic stimulus input masked by additive noise. The stimuli were pre-classified and banded into 100 classes based on the mean of each dimension. The network was trained to correctly classify a stimulus into the *i*^*th*^ class if the activity of the *i*^*th*^ readout neuron, described by a soft-max function, was greater than that of any other readout neuron given the same stimulus.

Initially, each neuron in a layer is fully connected to all neurons in the next layer, and the connection weight is randomly sampled from a uniform distribution *U*(0.01, 0.2). Each dimension of the stimulus vector acts as input to each neuron in the input (first) layer with predefined connection weights *W*_1_. The standard back-propagation learning algorithm (Materials and Methods) was used to train the network to classify the stimuli into the predefined 100 class labels. We divided the dataset into two parts, 60% of which were for training and 40% for testing. The network was exposed to all stimuli during each epoch to classify the stimuli, with 256 samples per training batch. We purposefully kept the model training and testing procedures to be sufficiently simple to more clearly identify the underlying mechanism for BTA emergence.

**Figure 2:**
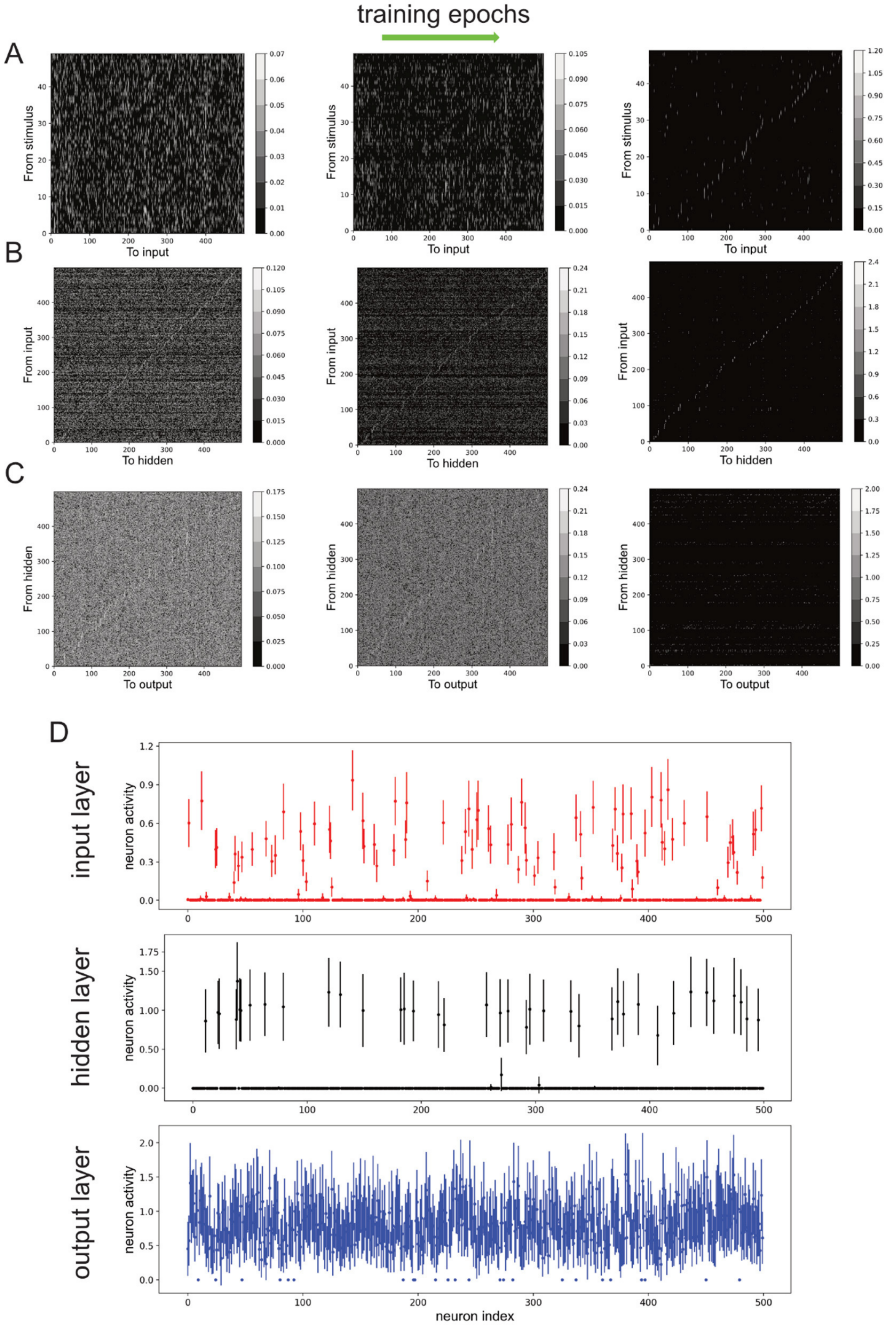
Neural network's non-negative connection weights and neuronal activities due to training. **(A–C)** Connection weights from stimulus to input neurons **(A)**, from input to hidden neurons **(B)**, and from hidden neurons to output neurons **(C)**, over training epochs (left to right). Color bars: values. **(D)** Neuronal activities of input, hidden and output neurons after training. Error bars: standard deviation across stimuli.

Figures 2A–C (left to right) shows snapshots of the evolution of the connection weights between the layers at the beginning, intermediate, and late stages during the training session. We can observe that as training progressed, the connection from stimulus-to-input layer *W*_1_, input-to-hidden layer *W*_2_, and hidden-to-output layer *W*_3_, became sparser while the strength of the remaining connections increased (Figures 2A–C). After 20 training epochs, the classification accuracy for the training dataset was 0.9957, while it was 0.9955 for the testing dataset.

Next, the activity of the neurons in the input, hidden and output layers (*h*_1_, *h*_2_ and *h*_3_) are investigated. As higher neuronal activity variability across different stimuli can indicate the encoding of more stimulus information, we used the standard deviation of neuronal activity as an indicator for neuronal responsiveness [mean neuronal activity values gave similar results (not shown)]. Specifically, by defining a neuron to be responsive or active if the standard deviation of its response to different samples was greater than 0.1, we found that the overall neuronal activities decreased after training, with the majority at the input and hidden layers being completely inactivated and not responding to the stimuli (Figure 2D). More precisely, at the end of 50 training sessions with 20 epochs per session (in last training epoch), there were 225.69 ± 15.67 active input neurons, 153.31 ± 20.79 active hidden neurons, and 498.85 ± 1.29 active output neurons. Note also the relatively small variations in the numbers of active neurons across different training sessions.

Compared to all other layers, the hidden layer had the least number of active neurons after training (Figure 2D, middle). This was also reflected in the lowest number of non-zero connection weights from the input layer to the hidden layer (Figure 2B, right). Thus, we have shown the emergence of BTA in the network using a simple learning procedure on a generic classification task. Next, we shall formally demonstrate that the BTA occurs only with non-negative connections.

### 3.2 Non-negative connectivity causes bow-tie architecture

**Figure 3:**
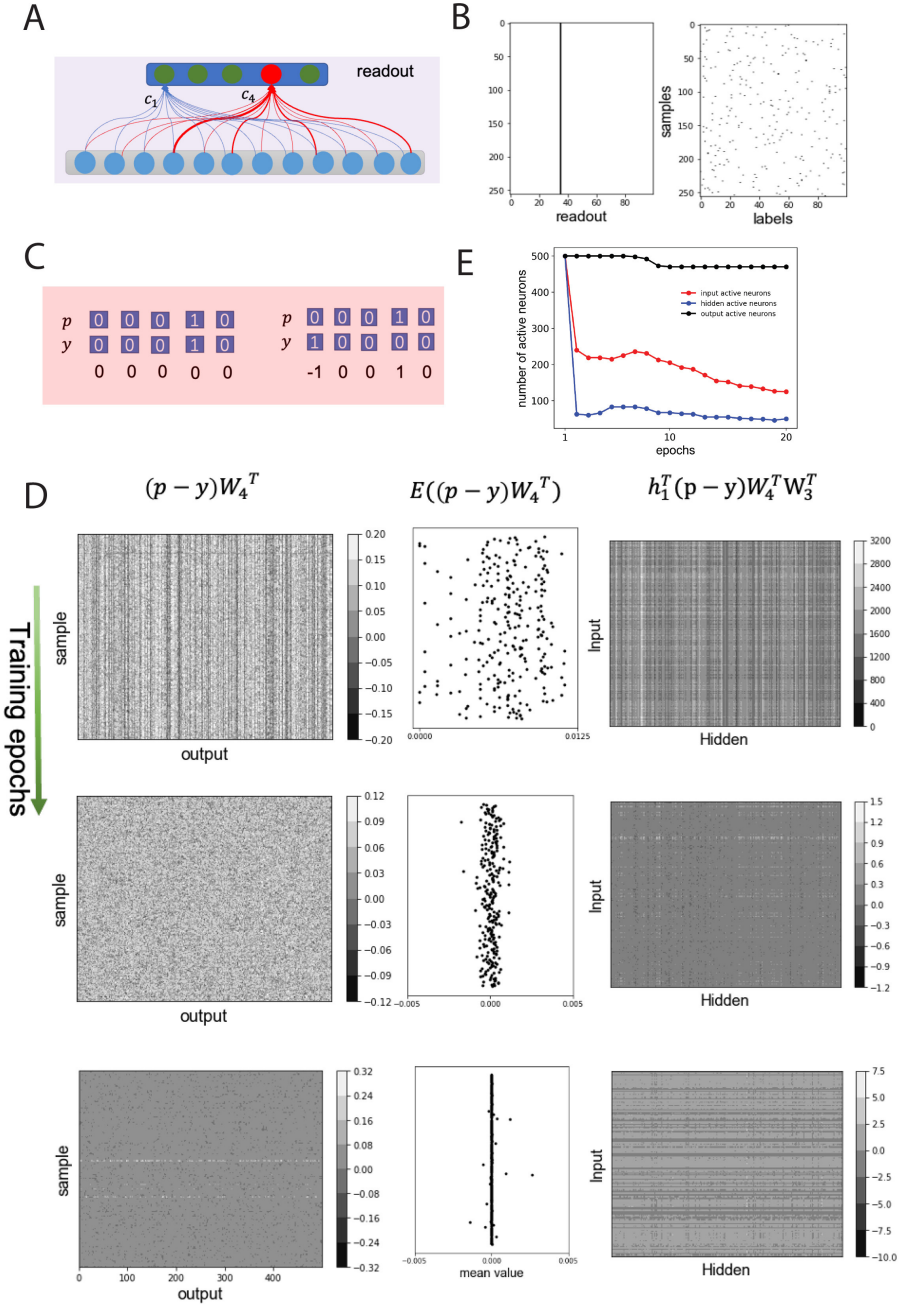
Non-negative connection weights amplify error signal and suppress neuronal activities, forming BTA. **(A)** Schematics of random initial connection weights from output to readout neurons. Weighted sum of afferents to readout neuron, c_4_ (red), larger than that of other neurons [e.g. neuron c_1_ (green)]. **(B)** Class labels for each sample in one batch (left) and neuronal activity in read-out layer (right) in the first training epoch (same decision made regardless of samples). **(C)** Examples of classification decisions *p*, actual class labels *y*, and their errors *p*−*y*. Left (right): correct (error) decision. **(D)** Backpropagation of error signals. A random batch chosen and error signal of samples illustrated. Top, middle, bottom rows: first, middle, final training epochs. Left (right) columns: errors backpropagated from readout to output (from hidden to input) neurons; middle column: mean backpropagated error values from readout neurons. *W*_3_ (*W*_4_): connections from hidden to output (output to readout) layer. *h*_1_: input layer's activity. **(E)** Number of active neurons in input (red), hidden (blue) and output (black) layers over training epochs.

The connection matrices from stimulus-to-input layer *W*_1_, input-to-hidden layer *W*_2_ and hidden-to-output layer *W*_3_ were initially set as random matrices. Thus, the activities of the output neurons can be considered as random variables, since the different samples were transformed by a series of random matrices. However, the weighted sum of afferent inputs to a readout neuron (e.g., neuron c_4_ in Figure 3A) from output neurons could initially just turned out to be larger than that of other readout neurons (e.g., neuron c_1_ in Figure 3A), and hence the network would have a strong tendency to produce the same readout decision with different sample classes, i.e., poor classification accuracy (~1%). As a consequence, the classification for all 256 samples in a batch in the first training epoch was concentrated to only one class (Figure 3B right panel).

Suppose for each sample, the readout layer of the neural network can be represented as a vector *p* (Figure 3C, top), and the predefined class label of a sample is represented as a vector *y* (Figure 3C, bottom) and randomly distributed in a batch (Figure 3B, left). Assuming that the network accurately classifies the *i*^*th*^ sample, then the *i*^*th*^ neuron in the output layer will be activated, and the *i*^*th*^ element of decision vector *p* will be 1. This means that the decision vector *p* is the same as the predefined label of the sample *y*, i.e., *p*−*y* = 0.

Then all elements of *p*−*y* are zero; thus, no error signal propagates back through the network layers, and the connection weights will not be changed. Otherwise, some elements of *p*−*y* are not zero. For example, Figure 3C (right) shows the 1^*st*^ and 4^*th*^ elements of *p*−*y* to be −1 and 1, respectively, because the reported class of the sample is 4 (i.e., *p*_4_ = 1) but the predefined class of the sample is 1 (i.e., *y*_1_ = 1). This error signal will then be back-propagated to the output layer, the hidden layer, and the input layer. In particular, the error signal back-propagated to the output neurons depends on the efferent weights from the output neurons to the readout neurons, which should be in the form of a vector as $\left(p-y\right)W_{4}^{T}$, where $W_{4}^{T}$ was the connectivity matrix for connections from output layer to readout layer (illustrated in Figure 3D, left column) (Rumelhart et al., 1986). Thus, the back-propagated error to the *m*^*th*^ output neuron is equal to the difference between the efferent weight from the *m*^*th*^ output neuron to the decision of the readout neuron (e.g., neuron *c*_4_ in Figure 3A) and the efferent weight from the *m*^*th*^ output neuron to the readout neuron should be activated as in the predefined label.

Often, the total efferent weights from the output neurons to the activated readout neuron were greater than those to the inactive readout neurons, so the error signal induced by a probe should be non-negative. Considering $\left(p-y\right)W_{4}^{T}$ for each sample in the batch data as a random variable, the expectation value of $\left(p-y\right)W_{4}^{T}$ should be greater than zero for incorrect classification, or equal to zero for correct classification (Figure 3D, middle column). According to the learning rule for connections from the hidden layer to the output layer, $W_{3}\leftarrow W_{3}-\eta h_{2}^{T}\left(p-y\right)W_{4}^{T}$ (Materials and methods, Equation 2), with learning rate η and hidden layer (layer 3) activity *h*_2_, the total connection weight from the hidden layer to the output layer should decrease. Due to the non-negative connectivity constraint *W*_2_ and *W*_3_ for the earlier layers, the expectation of $\eta h_{1}^{T}\left(p-y\right)W_{4}^{T}W_{3}^{T}$ should be positive, and the error signal *p*−*y* being amplified by positive connections.

Further, the weights of the connections from the input to the hidden layer, *W*_2_, decreased according to the learning rule $W_{2}\leftarrow W_{2}-\eta h_{1}^{T}\left(p-y\right)W_{4}^{T}W_{3}^{T}$. Considering that the stimulus is *s*∈[0, 1], the changed values of *W*_1_, i.e. $\eta s^{T}\left(p-y\right)W_{4}^{T}W_{3}^{T}W_{2}^{T}$, were usually smaller than that of *W*_2_. As a result, the connection weights from the input to the hidden layer, and from the hidden to the output layer decreased during the first training epoch.

Due to the decrease in the connection weights, the activity of the input and hidden neurons decreased significantly, and a portion of the input and hidden neurons became inactive in subsequent training epochs (Figure 3E). As training progressed, the back-propagated error signals decreased (Figure 3D, left and middle columns) and the magnitude of the weight change also decreased (Figure 3D, right column), implying that the inactive neurons in the input and hidden layers remained unchanged (Figure 3E; see also Figure 2D). Considering that deafferentation can lead to the degeneration of the postsynaptic neurons in sensory systems (Jones and Marc, 2005; Mazzoni et al., 2008; Kujawa and Liberman, 2009), the inactive neurons in the system will degenerate and can be removed from the system. Therefore, the non-negative weights between layers significantly reduced the number of active neurons in the hidden layer, implying that the BTA had emerged from the network.

To further test the role of non-negative connectivity constraint on BTA formation, we trained a network with identical architecture and on the same classification task as above, but without the non-negative weight constraint. We first found that the trained network could perform the classification task as well, albeit with slightly better classification accuracy (0.9984 for the training dataset and 0.9964 for the testing dataset). We also found that when using a non-negative connectivity constraint, the network's learning speed was slightly reduced. Particularly, if we defined the accuracy after the *n*^*th*^ epoch of training as *acc*(*n*), and the convergence rate α (Senning, 2007) of the network with and without non-negative connectivity constraints training are 0.850 and 0.825, respectively. Meanwhile, the trained connections between the layers were more dense without non-negative connectivity constraint , and neurons in the input, hidden and output layers were almost active compared to Figure 2D). Therefore, the training of the network without non-negative connectivity constraint did not result in the formation of BTA in the network.

In summary, the non-negative connection weights between network layers amplified the error signal and suppressed the activities of a significant number of neurons in the input layer and especially in the hidden intermediate layer, resulting in the emergence of BTA. Moreover, the classification accuracy and training speed achieved by the non-negative connectivity constrained network, which resulted in BTA, was comparable to that of the network without such constraint.

### 3.3 Bow-tie architecture is efficient, robust and generalizable

Since each synaptic connection is associated with metabolic cost (Attwell and Laughlin, 2001; Laughlin et al., 1998), we can evaluate the wiring cost for a neural network with BTA. For simplicity, we defined the total wiring cost of the connections as the sum of the absolute values of the network's connection weights and then compared the wiring cost for a network with and without non-negative connectivity constraints. It is clearly shown that the network with non-negative connectivity constraint had a much lower (about a third lower) connection cost than the one without this constraint (all *p*′*s* < 0.001). In addition, the number of active neurons in the input and the hidden layers of the network with BTA is much smaller than that in the network without BTA. These results suggest that networks with BTA may be highly energy efficient.

Although it may be desirable to have the BTA network to having a much smaller number of hidden neurons than other network layers, the hidden layer with its smaller structure may perhaps be more vulnerable to disruption or perturbation. To test the BTA's robustness with respect to structural changes, we separately trained 10 networks with identical initial architecture but different initial connection weights to perform the same classification task. We then randomly removed active neurons in the input, hidden, and output layers of the trained network.

**Figure 4:**
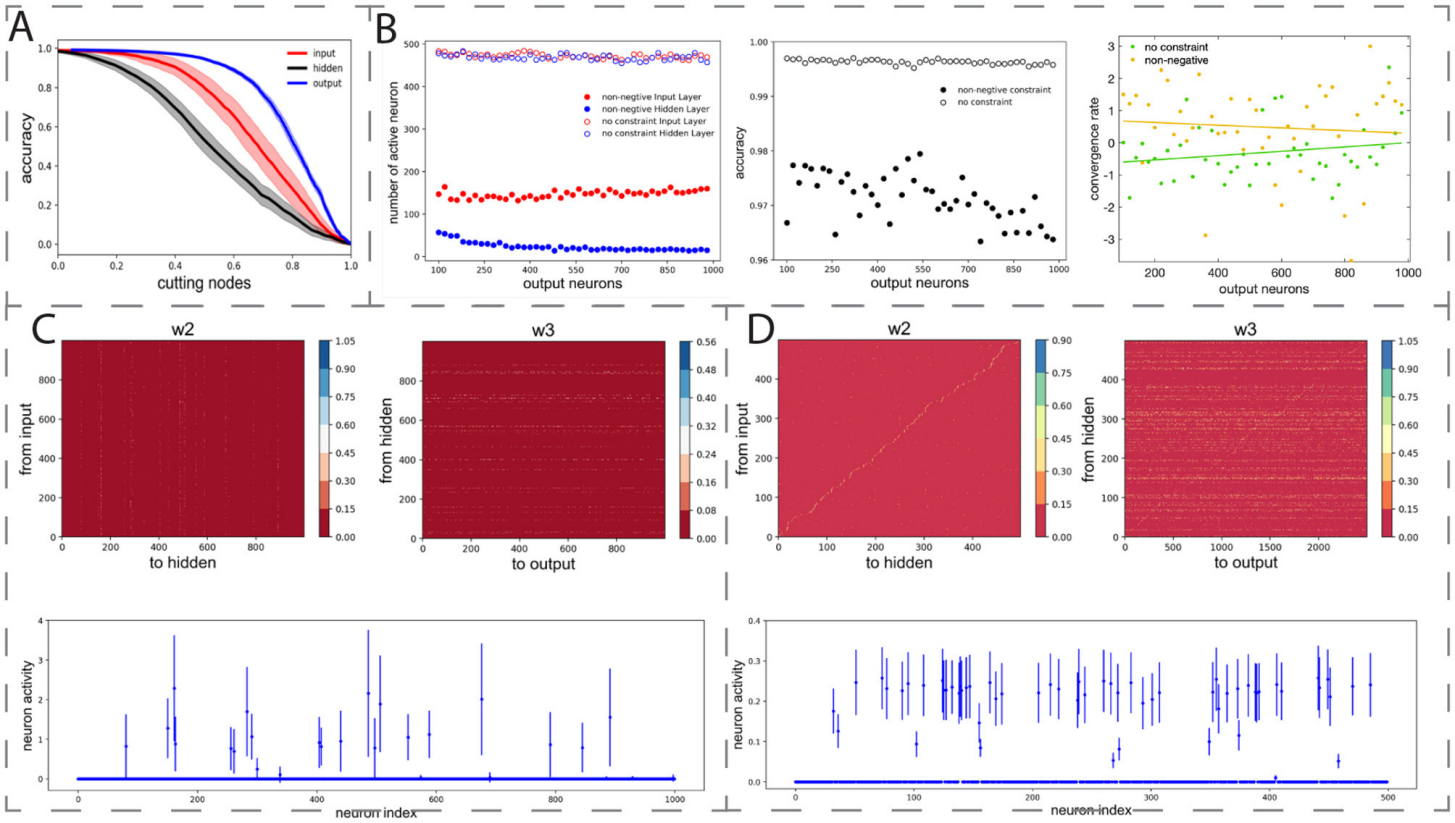
Robustness and generalizability of BTA. **(A)** Removal of neurons in the trained network at different layers decreased classification accuracy at different rates, with hidden neurons as the largest contributors. Horizontal axis: proportion of removed active neurons in input, hidden, or output layer. Shades: standard deviation of classification accuracy for 10 separately trained networks with different initial connection weights. **(B)** Left: with non-negative connectivity constraint, the number of output neurons did not substantially affect the final number of active input neurons (filled red circles) but decreased the active hidden neurons (filled blue circles). BTA was stabilised with increasing number of output neurons. Unfilled circles: without non-negative connectivity constraint. Middle, right: Classification accuracy (middle) and convergence rate (right) of networks with (yellow) and without (green) constraint. Fitted lines: linear regression *y* = 6.71 × 10^−4^*x*−0.67 (green); *y* = −4.23 × 10^−4^*x*+0.72 (yellow). **(C)** MNIST classification task. Trained connection weights from input to hidden layer (left) and from hidden to output layer (right) led to BTA. Small number of hidden neurons active after training (bottom). **(D)** Olfactory discrimination task. Trained connection weights from input to hidden layer (left), and from hidden to output layer (right) led to BTA. Small number of hidden neurons active after training (bottom).

The removal of neurons generally degrades the classification accuracy. In particular, for the same proportion of active neurons removed in the input, the hidden, and the output layers, we found that removing the hidden neurons degraded the classification accuracy (Figure 4A, black) more than that in the input or output layer (Figure 4A, red and blue). This was expected because the number of active hidden neurons in the trained network was much smaller than the number of active input and output neurons. Moreover, the small number of hidden neurons may encode key latent features, and their removal can be costly to performance. Thus, active hidden neurons act as a critical network hub for processing important information, and their removal has greater effect than removing neurons from the other layers.

As observed in Figure 3E that the model's active neurons in the output layer typically did not change relatively much, we next allowed the number of neurons in the output layer to vary from 100 to 1, 000, while fixing 500 input neurons and 500 hidden neurons in the network. The non-negative connectivity constraint was applied and each network structure was trained to perform the same classification task. We found that as the number of output neurons increased, the number of active input neurons did not vary too much, hovering around 140 (Figure 4B, left, filled red circles), but the number of active hidden neurons was substantially reduced (Figure 4B, left, filled blue circles). The BTA then became stabilized with further increase in the number of output neurons. Furthermore, increasing the number of output neurons did not have a substantial effect on the accuracy of classification accuracy (Figure 4B, middle) and the convergence rate of the training process (Figure 4B, right). Repeating the procedures on networks without the non-negative connectivity constraint did not show substantial changes in the number of input and hidden neurons (Figure 4B, left, opened filled circles). These results indicate that the BTA is robust to the variation in the number of output neurons, as had been observed in neural systems (Jang et al., 2020; Prochazka et al., 2014).

So far, we have made use of a generic classification task using synthetically generated dataset. Hence, our next step was to investigate whether the emergence of BTA can be generalized to various more realistic classification tasks. Thus, we first trained a network with non-negative connectivity constraint, initially with 1000 input neurons, 1, 000 hidden neurons, and 1, 000 output neurons to classify 10 handwritten digits in the well-known MNIST database with 60, 000 samples (LeCun et al., 2002). We found the classification accuracy on the training and testing datasets to be high, at 0.9613 and 0.9260, respectively. We then repeated the training on the MNIST data for 50 networks, and found the numbers of active neurons were on average 380.14 ± 15.80, 87.92 ± 7.35, and 997.76 ± 1.3 for the input, hidden, and output layers, respectively. Hence, after training, the connections became more sparse (Figure 4C) and the active neurons formed a BTA, similar to our observation with our synthetic generic dataset. Thus, the non-negative connectivity constrained network could classify very well with a robust emergent BTA.

Finally, we seek to know whether the BTA network can also perform odor discrimination task. Here, we trained the network with the same structure as in (Wang et al. 2021) but with a non-negative connectivity constraint to classify the 1, 008, 192 odor samples into 100 classes. This required a four-layer neural network, initially with 500, 500, 2, 500 and 100 neurons from the first odor stimulus layer to readouts (Wang et al., 2021). The accuracy of the training and testing datasets were found to be 0.9248 and 0.7865, respectively. After training, the network with non-negative connectivity constraint formed a BTA with sparse connectivity from input to hidden and from hidden to output neurons (Figure 4D). Based on 50 sessions of training with 20 epochs per session, we found that the numbers of active neurons were 62.08 ± 3.71, 48.97 ± 4.19 and 2, 308.74 ± 39.60 for the input, hidden, and output layers of the network, respectively. This was again a BTA, consistent with previous work (Wang et al., 2021). Overall, we have shown that BTA emerged even with different, more realistic tasks, suggesting that BTA emergence could be generalized and applied to different cognitive tasks.

## 4 Discussion

In this work, we provide the first evidence that non-negative (excitatory) connectivity serves as a mechanistic driver for the spontaneous emergence of bow-tie architecture (BTA) in neural networks. Unlike previous studies that rely on linear assumptions or predefined structures, our results show that training neural networks with non-negative weights on noisy signal classification tasks leads to the self-organization of BTA. This occurs through the amplification of back-propagated error signals and suppression of neuronal activity, particularly within the hidden layer (Figure 1). These findings are consistent with neurobiological observations showing a dominance of long-range excitatory over inhibitory projections (Douglas and Martin, 2004; Stepanyants et al., 2009), reinforcing the biological plausibility of our model and revealing a novel computational role for non-negativity in shaping neural architecture.

In contrast to the models used in previuos studies, neural networks are nonlinear, have complex connectivity, can perform cognitive tasks such as sensory discrimination, and can modify their architecture on a relatively faster timescale through learning (Martin et al., 2000). Our work complements and extends these studies by focusing on dynamic, learnable neural networks constrained by feed-forward non-negative connection. In contrast to (Tishby et al. 2000)'s theoretical framework, where compression is externally optimized, our networks spontaneously self-organize into BTA-like structures as a direct outcome of constrained learning dynamics. Compared with Friedlander's evolution models (Friedlander et al., 2015), our system demonstrates that BTA can emerge rapidly within task-optimized nonlinear networks, without predefined objective matrices or domain-specific structures. In this sense, our findings provide a more mechanistic and biologically motivated explanation for BTA emergence, grounded in modern learning theory. Moreover, instead of using a predefined neural network (Wang et al., 2021), we trained the network using a flattened architecture and found that BTA can emerge through training.

Moreover, as we have demonstrated, the non-negative constraint may play a potential role in promoting the emergence of a bow-tie architecture by encouraging sparse neural activity in the hidden layer. During training, we observed a notable reduction in the number of active neurons, which suggests that the network gradually converges toward more efficient internal representations. This observation is reminiscent of principles in biological systems, where functional efficiency is often associated with sparse and specialized neural coding strategies (Fakhar et al., 2025). Such sparsity may reflect an adaptive mechanism that enables the brain to maintain high-level cognitive function while minimizing metabolic cost. However, it is also important to note that excessive reduction in neural activity or synaptic connectivity may have pathological consequences. For example, synaptic pruning caused by immune dysfunction that exceeds functional thresholds has been implicated in neuropsychiatric disorders (Keshavan et al., 2020; Li et al., 2025). Therefore, while sparsity and non-negativity may support the emergence of efficient architectures like the bow-tie, they must be balanced to preserve robustness and functional integrity. This trade-off between efficiency and resilience may be an important consideration for future biologically inspired modeling studies.

Throughout training, we observed that the number of active neurons in the hidden layer decreased, eventually stabilizing. A dynamic we visualized in Figure 3E. This progressive sparsification suggests the development of efficient internal representations as learning proceeds. Such dynamics mirror sparse coding strategies seen in biological systems (Fakhar et al., 2025). The BTA models exhibited lower wiring cost, fewer active neurons, and faster convergence, without significantly compromising accuracy. These characteristics are in line with biological neural circuits, which often exhibit sparse coding and minimal energy expenditure while maintaining task performance (Babadi and Sompolinsky, 2014). Given the dynamic and heterogeneous nature of biological networks, we further examined the resilience of the learned BTA architectures. Numerical experiments showed that performance was most sensitive to disruptions in the bottleneck (hidden) layer (Figure 4A). This aligns with the functional importance of bottleneck regions such as the thalamus or neuromodulatory nuclei in the brain, which act as key integrative hubs and may be particularly vulnerable in disease contexts (Dorocic et al., 2014; Ogawa et al., 2014; Lee and Dan, 2012; Halassa et al., 2014).

We validated our approach across multiple classification tasks and class sizes–from 10 classes in digit recognition to 100 classes in synthetic signal and odor discrimination. BTA formation emerged consistently, independent of the task domain. Sensitivity analyses (Supplementary Section S1) confirmed that this effect holds robustly across different random seeds, network sizes, and pruning conditions. In particular, sparsity patterns in the hidden layers remained reproducible, and bow-tie formation proved stable even under architectural perturbation. To further assess generalization beyond the training distribution, we conducted out-of-distribution (OOD) testing (Supplementary Section S2). Models retained strong performance under moderate noise (accuracy 86%) but showed reduced accuracy with structured occlusion (accuracy 50%), suggesting a reliance on compressed, essential features–consistent with BTA's theoretical function as an information bottleneck. These findings reinforce the biological and computational relevance of the observed architecture.

### 4.1 Limitations and future directions

Our findings establish non-negativity as a key constraint for BTA emergence, with functional benefits and task generality. Future work should extend this framework to recurrent networks and inhibitory interactions to better mimic biological circuits. While these results offer important insights, several limitations highlight directions for future research. First, the model relies on a specific learning algorithm and employs simple feedforward neural networks focused on sensory discrimination tasks. Given that error back-propagation is not biologically plausible, future work should explore more biologically grounded learning algorithms. Second, while we investigate the bow-tie structure across layers, important biological features such as Dale's principle and recurrent connections are not incorporated. Additionally, although non-negative connections between layers may contribute to information compression, engineering constraints and real-world complexities are not addressed in this study. These simplifications enable theoretical tractability and clearer explanations of how non-negative connectivity can give rise to BTA. Considering the brain's complexity, where each neuron plays a specialized role in problem-solving, future research will aim to integrate more biologically plausible connectivity rules and neuronal dynamics to tackle more complex engineering challenges (Li et al., 2022; Davari et al., 2024; Gupta, 2024; Li et al., 2021). Furthermore, subsequent work will extend beyond the current model's scope by exploring whether the principles of BTA formation identified here generalize to other neural network architectures, alternative learning rules, and diverse cognitive functions.

## 5 Conclusion

In summary, this study identified non-negative (excitatory) connectivity as a key mechanistic driver of the spontaneous emergence of bow-tie architectures (BTA) in neural networks. By constraining network weights to be non-negative reflecting a biologically plausible condition. we showed that neural networks self-organize into compressive structures that amplify error signals while suppressing hidden-layer activity. These dynamics naturally lead to the formation of efficient bow-tie configurations. We further demonstrated that the emergent BTA architecture confers distinct functional advantages. Compared to unconstrained networks, BTA networks achieved reduced wiring cost, enhanced robustness to scaling, and improved task generalizability. Importantly, these properties were not limited to a specific task or architecture. As shown in our cross-dataset evaluations, BTA emergence generalized across multiple classification problems and output dimensionalities.

By providing a mechanistic account of BTA formation under biologically inspired constraints, our findings bridge observations in neuroscience with principles of artificial learning systems. This work not only offers insights into the computational organization of the brain, but also suggests strategies for designing more efficient and scalable machine learning models.

## Data Availability

The source code and implementation details are publicly available on GitHub: https://github.com/lzf531/Bow-tie-structure.
