# Supplementary material for: Non-negative connectivity causes bow-tie architecture in neural circuits
Source: Front Neural Circuits. 2025 Aug 18;19:1574877. doi: 10.3389/fncir.2025.1574877 (PMC12399558; doi:10.3389/fncir.2025.1574877)
Supplement: Supplementary file 1 [file Data_Sheet_1.pdf]

Supplementary Material

S1. Sensitivity Analysis Across Seeds, Network Sizes, and Pruning Conditions

To assess the robustness of bow-tie emergence and associated performance, we conducted a systematic sensitivity analysis across four random seeds (42, 123, 290, 2024), five network sizes (100-100-100 to 2000-2000-2000), and pruning settings (with/without pruning). We report model accuracy and hidden layer activation levels to characterize performance and structural bottlenecking.

S1.1 Accuracy Stability Across Seeds

Table 1 shows the mean accuracy and standard deviation across different seeds for each model size and pruning condition. The accuracy remained highly stable across seeds, with variance typically below 10^{-4}, indicating minimal dependence on initialization.

Table 1: Mean accuracy and standard deviation across seeds for each model size (RandomUniform initialization).

Model Size	Pruned	Mean Accuracy	Std Dev
100-100-100	No	0.9827	0.0017
	Yes	0.9823	0.0010
200-200-200	No	0.9905	0.0013
	Yes	0.9905	0.0011
500-500-500	No	0.9908	0.0011
	Yes	0.9906	0.0014
1000-1000-1000	No	0.9865	0.0025
	Yes	0.9860	0.0022
2000-2000-2000	No	0.9802	0.0022
	Yes	0.9784	0.0027

S1.2 Pruning and Neuron Activation Patterns

The effect of pruning on sparsity is summarized in Table 2. Pruning typically reduced the number of active neurons in the second hidden layer, which aligns with the emergence of a

functional bottleneck. Importantly, pruning had only marginal effects on accuracy, especially for moderate-sized networks.

Table 2: Average active units per hidden layer across seeds, with and without pruning.

Model Size	Pruned	Layer 1	Layer 2	Layer 3
100-100-100	No	100	98	99
	Yes	100	97	99
200-200-200	No	157	115	192
	Yes	156	115	196
500-500-500	No	146	53	478
	Yes	148	54	476
1000-1000-1000	No	162	38	962
	Yes	162	38	966
2000-2000-2000	No	223	22	1906
	Yes	206	24	1916

S1.3 Summary of Observations

- Accuracy is stable across seeds, confirming high reproducibility.
- Pruning does not significantly degrade accuracy, but enhances sparsity, particularly in the second hidden layer.
- Even without pruning, non-negativity alone leads to a bottleneck in hidden activity, consistent with bow-tie emergence.
- Peak performance occurs in medium-sized networks (e.g., 200–500), indicating an optimal tradeoff between capacity and efficiency.

S1.4 Implications for Bow-Tie Formation

These findings support the central claim of our work: **non-negative constraints alone are sufficient to induce bow-tie architectures** across a range of settings, and this effect is **robust to variation in seed, size, and pruning**. Furthermore, the resulting architectures are not only sparse and efficient, but also maintain high classification accuracy—highlighting the computational advantages of the emergent structure.

S2. Out-of-Distribution (OOD) Testing

To evaluate the model's generalization capacity under distributional shifts, we conducted out-of-distribution (OOD) tests using two perturbation strategies applied to the test set:

- **Gaussian noise:** We added additive Gaussian noise to the test inputs with a standard deviation of 0.3, simulating noisy sensory environments.

- **Input occlusion:** We randomly occluded (set to zero) 30% of input features for each test sample, simulating missing or corrupted inputs.

Both perturbations were applied to the same held-out test set used during model validation. The results are summarized below:

**OOD Condition**	**Accuracy**	**Loss**
Gaussian noise (std = 0.3)	0.8600	0.5754
Input occlusion (30%)	0.5003	1.8842

Table 3: Performance of the non-negative model under out-of-distribution (OOD) perturbations.

These results reveal that the trained non-negative network is relatively robust to unstructured additive noise, maintaining a high accuracy of 86% under Gaussian perturbation. In contrast, the same model performs poorly when a structured portion of the input is missing, with accuracy dropping to approximately 50% and the loss increasing substantially. This asymmetry suggests that the network relies on a sparse and possibly non-redundant set of input features, consistent with the observed bow-tie compression pattern in hidden layers. It also underscores the importance of input diversity and redundancy for achieving robustness to structured perturbations.
